# Brain Structural and Functional Reorganization in Tinnitus Patients Without Hearing Loss After Sound Therapy: A Preliminary Longitudinal Study

**DOI:** 10.3389/fnins.2021.573858

**Published:** 2021-03-11

**Authors:** Qian Chen, Han Lv, Zhaodi Wang, Xuan Wei, Pengfei Zhao, Zhenghan Yang, Shusheng Gong, Zhenchang Wang

**Affiliations:** ^1^Department of Radiology, Beijing Friendship Hospital, Capital Medical University, Beijing, China; ^2^Department of Otolaryngology Head and Neck Surgery, Beijing Friendship Hospital, Capital Medical University, Beijing, China

**Keywords:** tinnitus without hearing loss, structural and functional reorganization, voxel-based morphometry, independent component analysis, clinical variables

## Abstract

Sound therapy is one of the most common first-line treatments for idiopathic tinnitus. We aimed to investigate the brain structural and functional alterations between patients with idiopathic tinnitus without hearing loss (HL) and healthy controls (HCs) and between patients before and after sound therapy (narrow band noise). Structural and resting-state functional images were acquired from 13 tinnitus patients without HL and 18 HCs before and after 6 months of narrow band sound therapy (only patients received the treatment). Voxel-based morphometry (VBM) and independent component analysis (ICA) were conducted to separately investigate the brain structural and functional changes. Associations between brain changes and clinical variables were also performed. After the treatment, the % improvement of THI score was −1.30% (± 63.40%). Compared with HCs, tinnitus patients showed gray matter and white matter atrophy in the left middle temporal gyrus at baseline, and the gray matter volume was further reduced after the treatment. The patients also showed increased white matter volume in the cingulum (cingulate), right calcarine, left rolandic operculum, and left parietal and frontal lobes. Additionally, compared with HCs, tinnitus patients exhibited positive [medial visual network (mVN) and sensorimotor network (SMN), mVN and auditory network (AN)] and negative [mVN and lateral visual network (lVN)] internetwork functional connectivity (FC) at baseline and negative [left frontoparietal network (LFPN) and dorsal attention network (DAN), AN and posterior default mode network (pDMN)] internetwork FC after the narrow band sound therapy. The patients also showed negative [LFPN and right frontoparietal network (RFPN), LFPN and RFPN, anterior default mode network (aDMN) and AN, aDMN and DAN] internetwork FC after the treatment when compared with baseline. Our findings suggest that although the outcomes of idiopathic tinnitus patients without HL were not very good when the improvement of THI scores was used as an evaluation indicator, the patients experienced significant differences in auditory-related and non-auditory-related brain reorganization before and after the narrow band sound therapy, that is, sound therapy may have a significant effect on brain reorganization in patients with idiopathic tinnitus. This study may provide some new useful information for the understanding of mechanisms underlying idiopathic tinnitus.

## Introduction

Tinnitus is a phantom sound without an external source, which affects 10–15% of the adult population, and it seriously affects the quality of patients’ lives (Sereda et al., 2018). Prior studies have shown that tinnitus is caused by alterations in the brain (Eggermont and Roberts, 2004) and is associated with specific learning processes allowing increased awareness and continuous appraisal (i.e., tinnitus sensitization and centralization) (Zenner and Zalaman, 2004; Noreña and Farley, 2013). Moreover, many studies to date have shown significant brain structural and functional remodeling in tinnitus patients (Chen et al., 2015, 2020, 2021; Ryu et al., 2016; Schmidt et al., 2017). Therefore, it is very important to reverse tinnitus-related abnormal brain neural activity or reorganization.

To date, many treatment modalities have been applied to tinnitus patients, such as drug therapy, cognitive behavioral therapy (CBT), tinnitus counseling, cochlear implants (CIs), tinnitus retraining therapy, hearing aids, brain stimulation, and sound therapy (Langguth et al., 2013; Zenner et al., 2017; Sereda et al., 2018; Bae et al., 2020). There is no satisfying treatment that can benefit all patients (Langguth et al., 2013). However, of the abovementioned treatment methods, sound therapy, such as using a noise generator with an unmodulated frequency or involving the use of a recorded noise or a special noise source or mask device (Jastreboff, 1999; Oishi et al., 2013), has been widely suggested for the management of tinnitus in many studies (Jastreboff, 1999; Oishi et al., 2013; Han et al., 2019a,b; Lv et al., 2020). Meanwhile, according to meta-analytic evidence (Sereda et al., 2018), sound therapy is the first-line treatment method in UK audiology departments for tinnitus patients (together with hearing aids and information and advice) (Hobson et al., 2012; Hoare et al., 2014; Sereda et al., 2015; Tutaj et al., 2018) and was even listed as an option in the clinical guidelines (Henry et al., 2002; Tunkel et al., 2014). During this treatment, the generated sound will be set based on tinnitus features, including its pitch, loudness, and minimum masking level. This sound reduces the contrast between the tinnitus and the environment, diminishes sensitivity to tinnitus, and promotes habituation to the tinnitus sensation (Makar et al., 2017). Narrowband noise is the most commonly used and effective sound therapy method (Henry et al., 2002). However, to the best of our knowledge, few studies to date have explored the effect of sound therapy on brain remodeling in idiopathic tinnitus. Almost all studies have focused only on alterations in brain function after treatment. These studies have suggested that sound therapy achieved good outcomes when taking improvements in tinnitus handicap inventory (THI) scores as an evaluation standard (Han et al., 2019a,b; Lv et al., 2020). For example, Han et al. (2019b) and Lv et al. (2020) have indicated that sound therapy significantly changed or even had a normalizing effect on the abnormal functional connectivity (FC) in the brains of patients with idiopathic tinnitus and that the changed FC was correlated with the severity of tinnitus.

Moreover, although there are many studies on brain structural and functional changes in tinnitus patients at present (Aldhafeeri et al., 2012; Seydell-Greenwald et al., 2014; Chen et al., 2015; Ryu et al., 2016; Han et al., 2020), many of these subjects had different degrees of hearing loss (HL) (Aldhafeeri et al., 2012; Seydell-Greenwald et al., 2014; Ryu et al., 2016; Han et al., 2020). Although some researchers have noticed that HL may have an impact on the results, their studies were mainly on brain reorganization in patients without any treatment (Husain et al., 2011; Benson et al., 2014; Schmidt et al., 2017). To date, there are few studies on the changes in both brain structure and function in tinnitus patients without HL after sound therapy.

Therefore, based on the above studies, to explore the brain alterations between tinnitus patients and healthy controls (HCs) and between patients before and after sound therapy, in the present study, we combined voxel-based morphometry (VBM) and independent component analysis (ICA) in patients with tinnitus in the early stage (duration less than 48 months) without HL before and after sound therapy and further explored their relationships with clinical variables. We defined subjects with a duration of fewer than 48 months being in the early stage, which is consistent with our previous studies (Liu et al., 2018; Chen et al., 2020).

## Materials and Methods

### Participants

Thirteen patients with untreated persistent idiopathic tinnitus and 18 HCs were enrolled in this study. All the patients had persistent idiopathic tinnitus, and the duration had been persistent for more than 6 months and less than 48 months, without any history of associated brain diseases confirmed by conventional magnetic resonance imaging (MRI), no preexisting mental or cognitive disorder, and no MRI contradictions. Tinnitus was present as a single high/low-pitched sound and 2 high/low-pitched sounds without any rhythm. All the patients were defined as tinnitus without HL based on audiogram results, which was defined as more than 25 dB HL at frequencies ranging from 250 to 8 kHz (0.250, 0.500, 1, 2, 3, 4, 6, and 8 kHz) in a pure-tone audiometry (PTA) examination. All the patients in this study were without pulsatile tinnitus, sudden deafness, Ménière’s disease, hyperacusis on physical examination, otosclerosis, and other neurological diseases. We asked all the patients to complete the THI (Newman et al., 1996) and a visual analog scale (VAS) to assess the severity of disease at the time of admission. We also evaluated the severity of depression and distress of the patients at that time. Moreover, we assessed the hearing of the HCs, and all of them had normal hearing. Other exclusion criteria for the tinnitus patients were also applied with the HCs.

The study protocol was approved by the institutional review board (IRB) of Beijing Friendship Hospital, Capital Medical University, Beijing, China. All the subjects were informed of the purpose of the study and gave written consent in accordance with the Declaration of Helsinki. The registration number of the study on ClinicalTrials.gov is NCT03764826.

### Sound Therapy and Clinical Evaluation

The sound therapy applied is a customized personal sound therapy based on patients’ tinnitus features. We used the special tinnitus therapeutic instrument: eMasker^®^ (Micro-DSP Technology Co., Ltd), which is a customized personal sound therapy device based on tinnitus characteristic test results. We advise patients to use it in a quiet environment to achieve the best therapeutic effect. First, to characterize the tinnitus and prepare for treatment, the audiologists in our group examined all the patients for tinnitus loudness matching, pitch matching, minimum masking level, and residual inhibition. Then, we applied narrow band noise (that was used for treatment) to treat tinnitus for 6 months, 20 min each time, three times per day. The loudness of sound we applied for each patient was set as 5 dB over the tinnitus loudness. The frequency was set as a 1 kHz narrow band while setting the tinnitus frequency as the middle point of the delivered sound (i.e., tinnitus frequency ± 0.5 kHz, for example, tinnitus frequency = 3 kHz, low sound cut = 2.5 kHz, high sound cut = 3.5 kHz). In this procedure, we used the THI scores to assess the severity of tinnitus before and after treatment. In our study, consistent with prior research, a reduction in THI scores to 16 points or a reduction of 17 points or more was considered effective treatment (Zeman et al., 2011). Therefore, we defined the primary outcome of this study as THI score changes. The HCs were not given any particular kind of sound exposure during the study. We also calculated ^Δ^ THI scores and improvement in THI scores in all patients with tinnitus, which were defined as follows: ^Δ^ THI score = THI on admission—THI follow-up; % improvement in THI score = (THI score at 6 months follow-up—THI score on admission) ÷ THI score on admission × 100%.

### Image Acquisition

Structural and functional imaging data of the idiopathic tinnitus patients at baseline (without any treatment) and after treatment (6 months) and from HCs were obtained using a 3.0T MRI system (Prisma, Siemens, Erlangen, Germany) with a 64-channel phase-array head coil. During the scanning process, we used tight but comfortable foam padding to minimize head motion and earplugs to reduce scanner noise. All the participants were asked to stay awake, close their eyes, breathe evenly, and try to avoid specific thoughts. We used a conventional brain axial T2 sequence before the structural and functional scans to exclude any visible brain abnormalities. Using a 3D magnetization-prepared rapid gradient-echo sequence (MP-RAGE), we obtained high-resolution three-dimensional (3D) structural T1-weighted images. The parameters were as follows: repetition time (TR) = 2,530 ms; echo time (TE) = 2.98 ms; inversion time (TI) = 1,100 ms; FA = 7°; number of slices = 192; slice thickness = 1 mm, bandwidth = 240 Hz/Px; field of view (FOV) = 256 × 256 mm^2^; and matrix = 256 × 256, resulting in an isotropic voxel size of 1 × 1 × 1 mm^3^. In addition, we also obtained resting-state functional images of all participants using an echo-planar imaging (EPI) sequence. The scanning parameters were as follows: 33 axial slices with a slice thickness = 3.5 mm and interslice gap = 1 mm, TR = 2,000 ms; TE = 30 ms; FA = 90°; bandwidth = 2,368 Hz/Px; FOV = 224 × 224 mm^2^; and matrix = 64 × 64. The latter parameters resulted in an isotropic voxel size of 3.5 × 3.5 × 3.5 mm^3^. The total number of volumes acquired was 240.

### Processing of Structural Images and Voxel-Based Morphometry Analysis

We performed postprocessing of the structural data using CAT12^1^ implemented in Statistical Parametric Mapping (SPM) software (version 12)^2^. SPM 12 was installed in MATLAB 2016a (Math Works, Natick, MA, United States). First, all the structural images were screened for movement artifacts. Next, the structural images were segmented into gray matter, white matter, and cerebrospinal fluid (CSF) areas using the unified standard segmentation option in SPM12. The individual gray matter and white matter components were then normalized into the standard Montreal Neurological Institute (MNI) space using the Diffeomorphic Anatomical Registration through Exponentiated Lie algebra (DARTEL) algorithm (Ashburner, 2007) after segmentation. The normalized gray matter and white matter components were modulated to generate the relative gray matter volume (GMV) and white matter volume (WMV) by multiplying by the non-linear part of the deformation field at the DARTEL step. The Gaussian kernel used to smooth the resulting GMV and WMV images was 6 mm full-width at half-maximum (FWHM).

### Preprocessing of Resting-State Functional Images

We used the batch-processing tool Data Processing and Analysis for (Resting-State) Brain Imaging (DPABI)^3^ (Yan et al., 2016) to preprocess the resting-state functional MRI (rs-fMRI) data, which is based on SPM12. First, to allow for steady-state magnetization and stabilization of the subject, we removed the first 10 volumes of each functional time series of all the participants. After that, we conducted slice timing correction on the remaining 230 volumes. Head motion between volumes was evaluated and corrected using rigid body registration, and we excluded datasets with maximum translation exceeding 2.5 mm, maximum rotation exceeding 2.5° or mean framewise displacement (FD) >0.3 (Yan et al., 2013). Next, based on the standard stereotaxic coordinate system, we spatially normalized the corrected fMRI images to an MNI template brain. Then, each voxel was resampled to isotropic 3 mm × 3 mm × 3 mm. After that, to remove the possible variances from the time course of each voxel (including the WM and CSF signal and Friston-24 head motion parameters), the 26 nuisance covariates were regressed out. Finally, the Gaussian smoothing kernel for the rs-fMRI images was a 6-mm FWHM.

### ICA Analysis

We performed ICA through GIG-ICA using GIFT software (version 3.0b)^4^. The main steps included data reduction, application of the ICA algorithm, and back-reconstruction for each subject. In the present study, we performed group independent component analysis (GICA) 100 times on tinnitus patients and HCs using 20 and 30 components separately. During this process, through visual inspection and previous reports (see the “Results” section for details), we identified nine components as meaningful resting-state networks (RSNs). We also obtained the individual-level components using back-reconstruction and transformed the subject-specific spatial maps to z scores.

### Intranetwork Functional Connectivity Analysis

The main process was consistent with a previous study (Chen et al., 2018). To generate a sample-specific spatial map for each component, each ICA component was entered into a random-effect one-sample *t*-test using a family wise error (FWE) correction (*p* < 0.05) with a cluster size of >100 voxels (Song et al., 2013; Wang et al., 2014). We compared the differences in intranetwork FC between the tinnitus patients and HCs at baseline and after treatment using a two-sample *t*-test and applied a paired *t*-test to compare intranetwork FC between patients before and after treatment [false discovery rate (FDR) corrected *p* < 0.05]. Using a general linear model (GLM), we extracted and compared intranetwork FC of each region of interest (ROI) with a significant difference between groups, with age and sex serving as covariates. For the ROI-based analyses, we used Cohen’s d (Parker and Hagan-Burke, 2007) to determine the effect size of each comparison.

### Internetwork Functional Connectivity Analysis

During the process of the internetwork FC analysis, first, by averaging the time courses of all voxels within the sample-specific RSN mask of each subject, we calculated the mean time course of each RSN. Then, we calculated Pearson’s correlation coefficients of the mean time courses between all pairs of RSNs for each subject and then converted them to *z*-values using Fisher’s r-to-z transformation to improve normality. For each group, individual z-values were entered into a random-effect one-sample *t*-test to determine whether the correlation between each pair of RSNs was statistically significant (*p* < 0.05). Intergroup comparisons were carried out only if the internetwork FC of each group was statistically significant (*p* < 0.05). We performed GLM with age and sex as covariates to determine whether the pairs of internetwork FC were significantly different (*p* < 0.05) between the patients and HCs at baseline and after treatments using a two-sample *t*-test and between the patients before and after treatment using a paired *t*-test.

### Statistical Analyses

During the statistical analysis process, we assessed all the data for normality using the Kolmogorov–Smirnov test. If the data were identified as not normally distributed, we applied non-parametric tests. First, we applied the voxel wise two-sample *t*-test and paired *t*-test in SPM12 to compare the whole-brain GMV and WMV differences between the tinnitus patients and HCs at baseline and after treatment and between the patients before and after narrow band sound therapy (voxel-level uncorrected *p* < 0.001, non-stationary cluster-level FWE correction with *p* < 0.05), and age and sex served as nuisance covariates. Next, the mean GMV, WMV, and FC values of each cluster that showed statistical significance were extracted for subsequent analyses. Then, we conducted a partial correlation analysis to explore any potential associations between brain alterations (intra/internetwork level) and clinical variables in tinnitus patients after removing age and sex effects (FDR *p* < 0.05). The last steps were performed using IBM SPSS Statistics version 23.0 (IBM Inc., Armonk, NY, United States).

GMV, WMV, and FC results were presented using MRIcron^5^, BrainNet Viewer^6^ (Xia et al., 2013) and xjView^7^.

## Results

### Demographic Data

Table 1 shows detailed demographic data of the 13 tinnitus patients with persistent idiopathic tinnitus characteristics and 18 HCs. We acquired the THI scores before and after narrow band sound therapy for all patients. In this study, the outcome of tinnitus patients was considered poor after sound therapy when taking THI score improvements as the evaluative measurement.

**TABLE 1:** Demographic and clinical data of the tinnitus patients and healthy controls.

**Demographic**	**Tinnitus (baseline, *n* = 13)**	**Tinnitus (6 months, *n* = 13)**	**Control (*n* = 18)**	***P*-value**
Age, years	42.23 (± 13.98)		45.33 (± 9.64)	0.470^a^
gender	6 males, 7 females		9 males, 9 females	0.561^b^
THI score	53.38 (± 28.10)	45.85 (± 25.16)	NA	0.379^c^
^Δ^ THI score	7.54 (± 29.78)		NA	NA
% improvement of THI score	−1.30% (±63.40%)		NA	
Duration, months	6 and ≤48		NA	NA
Type ^#^	7: 2: 3: 1		NA	NA
Tinnitus pitch	250∼8,000 Hz		NA	NA
Laterality	3 right, 4 left, 6 bilateral		NA	NA
Normal hearing	All		All	NA

**THI, Tinnitus Handicap Inventory, ^△^ THI score = THI _*baseline*_ – THI _*treated*_, NA: not applicable. ^*a*^Two-sample t-tests. ^*b*^Chi-square test. ^*c*^Paired-samples t-tests. ^#^1 single high-pitched sound vs. 1 single low-pitched sound vs. 2 high pitched sounds vs. 2 low pitched sounds.**

### Brain Structural Changes Between the Patients and HCs at Baseline and After Treatment and Between the Patients Before and After Treatment

**FIGURE 1:**
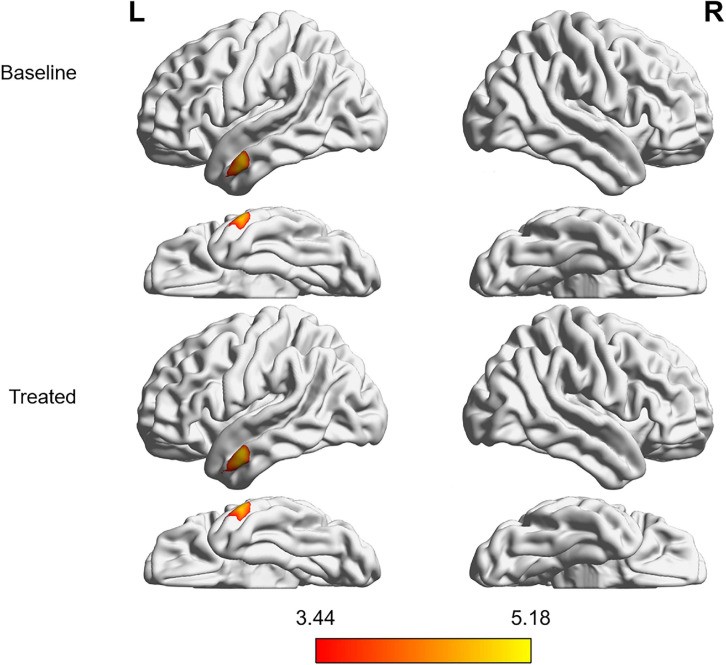
Intergroup differences in GMV between tinnitus patients without HL and HCs before and after sound therapy. At baseline, compared with the HCs, the patients with tinnitus without HL exhibited reduced GMV in the left MTG. After sound therapy (treated), the tinnitus patients continued to show decreased GMV in the same region, and the volume of gray matter was further reduced (corrected at the non-stationary cluster level with FWE *P* < 0.05). HL, hearing loss, HC, healthy control, MTG, middle temporal gyrus, FWE, family wise error, GMV, gray matter volume. The color bar represents the extent of reduction in GMV.

**FIGURE 2:**
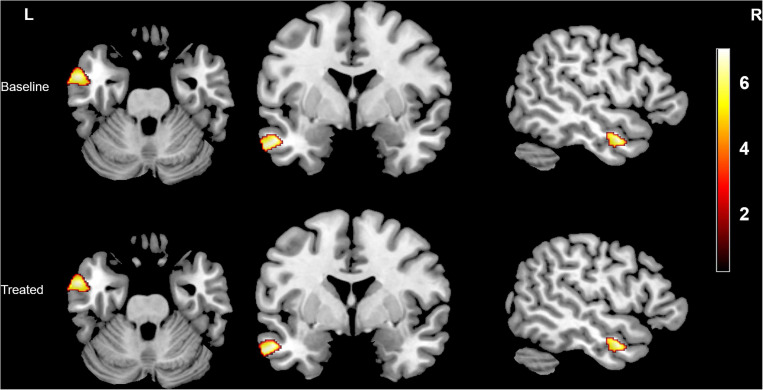
Intergroup differences in WMV between the tinnitus patients without HL and HCs before and after sound therapy. At baseline, compared with the HCs, the patients with tinnitus without HL exhibited reduced WMV in the left MTG (corrected at non-stationary cluster level with FWE *p* < 0.05). After sound therapy (treated), the brain region with decreased WMV was still the same area, but the trend in the WMV reduction had diminished (uncorrected). HL, hearing loss, HC, healthy control, MTG, middle temporal gyrus, FWE, family wise error, WMV, white matter volume. The color bar represents the extent of reduction in WMV.

**FIGURE 3:**
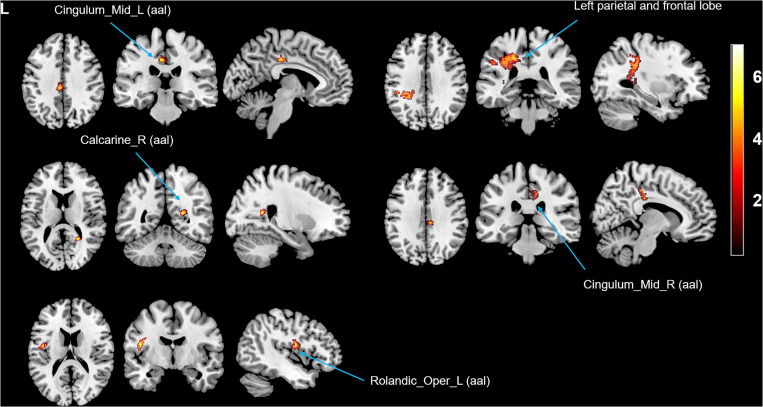
Intergroup differences in WMV between the tinnitus patients before and after sound therapy. Compared with baseline, after sound therapy, the patients with tinnitus exhibited increased WMV in the cingulum (cingulate), right calcarine, left rolandic operculum, and left parietal and frontal lobes (corrected at the non-stationary cluster level with FWE *p* < 0.05). FWE, family wise error, WMV, white matter volume. The color bar represents the extent of reduction in WMV.

Compared with the HCs, the tinnitus patients showed decreased GMV (Figure 1 and Table 2) and WMV (Figure 2 and Table 2) in the left middle temporal gyrus (MTG) at baseline, and the GMV was even further reduced after treatment (non-stationary cluster-level FWE correction with *p* < 0.05) (although the trend in the WMV reductions showed a decrease, the *P*-value was uncorrected). Additionally, the patients showed increased WMV in the cingulum (cingulate), left parietal and frontal lobes, right calcarine, and left rolandic operculum after treatment compared with the baseline (non-stationary cluster-level FWE correction with *p* < 0.05) (Figure 3 and Table 2).

**TABLE 2:** Difference in gray/white matter volume between the tinnitus patients and healthy controls and between patients before and after sound therapy.

**Anatomical region**	**MNI coordinate**			**Voxel**	**Peak**
				**size**	***T*-value**
	**x**	**y**	**z**		
**Gray matter (FWE cluster *p* < 0.05)**					
Gray matter volume (baseline)					
Tinnitus patients < Healthy controls					
Left middle temporal gyrus	−65	−3	−24	690	5.06
Gray matter volume (after treatment)					
Tinnitus patients < Healthy controls					
Left middle temporal gyrus	−65	−3	−23	831	5.18
**White matter**					
White matter volume (baseline)					
Tinnitus patients < Healthy controls					
Left middle temporal gyrus (FWE cluster *p* < 0.05)	−60	2	−29	373	5.39
White matter volume (after treatment)					
Tinnitus patients < Healthy controls					
Left middle temporal gyrus (uncorrected)	−60	2	−29	362	5.49
White matter volume (FWE cluster *P* < 0.05)					
Tinnitus patients (baseline) < Tinnitus patients (after treatment)					
Cingulum_Mid_L (aal)	−8	−27	39	110	8.40
Calcarine_R (aal)	24	−51	15	65	7.98
Cingulum_Mid_R (aal)	8	−30	44	115	6.50
Rolandic_Oper_L (aal)	−45	−6	15	113	6.06
Left parietal and frontal lobe	−29	−41	44	1,345	8.04

**MNI, Montreal Neurological Institute; FWE, family wise error; L, left; R, right.**

### Resting-State Network Functional Connectivity Changes Between the Patients and HCs at Baseline and After Treatment and Between the Patients Before and After Treatment

**FIGURE 4:**
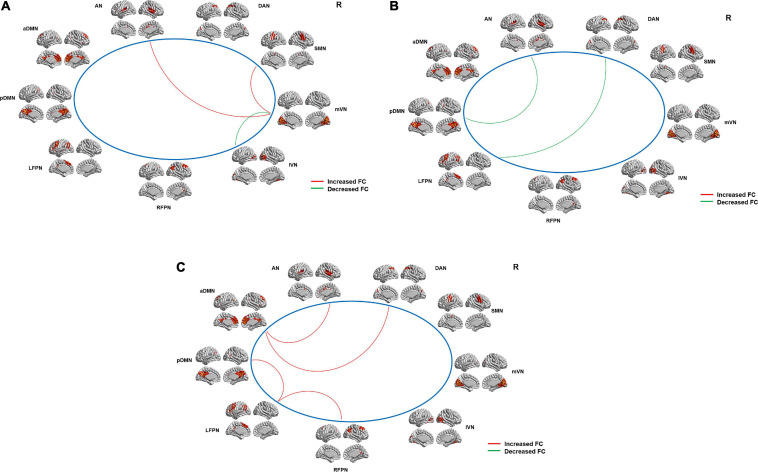
Intergroup differences in internetwork FC between the tinnitus patients and HCs and between the patients before and after sound therapy. **(A)** Compared with the HCs, the tinnitus patients exhibited a decreased (i.e., positive) (mVN and lVN) or an increased (i.e., less negative) (mVN and SMN; mVN and AN) internetwork FC at baseline. **(B)** Compared with HCs, tinnitus patients showed a decreased (i.e., positive) (AN and pDMN; DAN and LFPN) internetwork FC after sound therapy. **(C)** Compared with the patients at baseline, the tinnitus patients exhibited increased (i.e., negative) (LFPN and pDMN; LFPN and RFPN; aDMN and AN; aDMN and DAN) internetwork FC after sound therapy. AN, auditory network; aDMN, anterior default mode network; pDMN, posterior default mode network; LFPN, left frontoparietal network; RFPN, right frontoparietal network; lVN, lateral visual network; mVN, medial visual network; SMN, sensorimotor network; DAN, dorsal attention network; FC, functional connectivity, HC, healthy control. The red line represents positive FC; the green line represents negative FC.

The nine RSNs identified in our study were as follows: the auditory network (AN), the anterior (aDMN), and posterior (pDMN) default mode networks, the left (LFPN) and right (RFPN) frontoparietal networks, the medial (mVN) and lateral (lVN) visual networks, the sensorimotor network (SMN), and the dorsal attention network (DAN) (Figures 4A–C). The locations of these RSNs were in line with some prior studies (Mantini et al., 2007; Smith et al., 2009; Du et al., 2015; Ma et al., 2016).

### Altered Functional Connectivity Within and Between Resting-State Networks

We found that the tinnitus patients exhibited a decreased (i.e., positive) (mVN and lVN, *p* = 0.007) or an increased (i.e., less negative) (mVN and SMN, *p* = 0.018; mVN and AN, *p* = 0.035) internetwork FC at baseline when compared with HCs (Figure 4A and Table 3). Additionally, compared with the HCs, the patients showed a decreased (i.e., positive) (AN and pDMN, *p* = 0.008; DAN and LFPN, *p* = 0.005) internetwork FC after treatment (Figure 4B and Table 3). Meanwhile, tinnitus patients also exhibited an increased (i.e., less negative) (LFPN and pDMN, *p* = 0.023; LFPN and RFPN, *p* = 0.030; aDMN and AN, *p* = 0.037; aDMN and DAN, *p* = 0.008) internetwork FC after treatment compared with baseline (Figure 4C and Table 3). However, we did not observe any intranetwork FC changes between the patients and HCs at baseline or after treatment or between the patients before and after treatment within the nine RSNs.

**TABLE 3:** Intergroup differences in the inter-network functional connectivity.

**Functional**	**Baseline**		**Treated**		**Patients _pre_**	
		
**connectivity**	**(HCs-Patients)**		**(HCs-Patients)**		**– Patients _pos_**	
	***t***	***p***	***t***	***p***	***t***	***p***
mVN-SMN	−**2.511**	**0.018**	–0.285	0.778	0.511	0.614
mVN-AN	−**2.219**	**0.035**	0.842	0.407	0.219	0.829
mVN-lVN	**2.904**	**0.007**	0.637	0.529	–0.448	0.658
LFPN-pDMN	0.162	0.873	–0.150	0.882	−**2.404**	**0.023**
LFPN-RFPN	0.999	0.326	1.504	0.143	−**2.312**	**0.030**
aDMN-AN	–0.671	0.508	0.772	0.446	−**2.216**	**0.037**
aDMN-DAN	–0.044	0.966	–0.023	0.982	−**2.904**	**0.008**
pDMN-AN	0.500	0.621	**2.836**	**0.008**	0.218	0.830
LFPN-DAN	0.500	0.623	**3.033**	**0.005**	–0.079	0.937

**mVN, medial vision network; SMN, sensorimotor network; AN, auditory network; lVN, lateral network; LFPN, left frontoparietal network; RFPN, right frontoparietal network; aDMN, anterior default mode network; pDMN, posterior default mode network; DAN, dorsal attention network; HCs, healthy controls. The bold values indicate significant differences between the tinnitus patients and HCs at baseline and after sound therapy and the differences between the tinnitus patients before and after the treatment (p < 0.05).**

### Correlations Between the Brain Structural and Functional Changes and Extent of THI Score Changes

We performed partial correlations between the GMV, WMV, and internetwork FC values and the THI scores, ^Δ^ THI scores, % improvement in THI scores, and other clinical variables (such as duration and VAS scores) in patients with tinnitus after controlling for age and sex. We did not detect any associations among these variables (*p* > 0.05).

## Discussion

In the present study, all the patients had poor outcomes after the treatment when using improvements or changes in THI scores as the evaluation standard, which define a THI score reduced to 16 points or a reduction of 17 points or more as an effective treatment or good outcome (Zeman et al., 2011). Moreover, in the classic frequencies (from 250 – 8 kHz: 0.250, 0.500, 1, 2, 3, 4, 6, and 8 kHz), all the patients in this study were identified as tinnitus without HL, which is in line with one of our previous studies (Chen et al., 2020), although we cannot eliminate the possibility of hidden HL currently. Combining VBM and ICA analyses, we found significant brain gray/white matter atrophy in the auditory-related cortex at baseline, and the GMV was further reduced after sound therapy. Additionally, patients showed increased WMV in some regions that are not directly related to auditory function after treatment compared with baseline. More importantly, tinnitus patients showed significantly changes in internetwork FC in auditory-related and non-auditory-related networks at baseline or after sound therapy when compared with HCs or in the comparison of the patients before and after treatment. Therefore, although the idiopathic tinnitus patients group without HL with poor outcomes, narrow band sound therapy may have a significant effect on brain reorganization in patients with tinnitus. In this study, we chose narrow band noise as the applied sound therapy as it is a commonly used kind of sound therapy with high cost-effectiveness (Henry et al., 2002). It promotes habituation to the tinnitus sensation, diminishes the sensitivity of tinnitus, and reduces the contrast between tinnitus and the environment (Lv et al., 2020).

### Auditory-Related Brain Structural and Functional Alterations Between the Patients and HCs and Between the Patients Before and After Sound Therapy

The temporal gyrus is closely related to auditory function, especially the posterior MTG, which is part of the auditory primary cortex. Studies have shown that tinnitus can cause significant cortical changes in the MTG (Boyen et al., 2013; Pereira-Jorge et al., 2018). For example, Boyen et al. (2013) found that the change in gray matter in the auditory primary cortex (including the MTG) was correlated with tinnitus rather than with HL, and they speculated that the continuous sensation of an internal sound, such as the tinnitus percept, may cause the changes in the MTG. Consistent with previous studies, our findings suggested that tinnitus without HL can cause significant brain structural changes in the auditory cortex, although we cannot eliminate the possible effect of hidden or slight HL on the brain alterations. More interestingly, after the narrow band sound therapy, we found that the GMV in the MTG was further reduced, while the trend for further decreases in WMV in the same area was diminished, but the *P*-value with the WMV was uncorrected.

In addition to the auditory-related brain structural changes, we also observed increased (i.e., negative) internetwork FC between the AN and mVN in the patients with tinnitus than the HCs before treatment. The AN and VN are independent processing systems for auditory and visual functions. In tinnitus patients, the increased internetwork FC between the two processing networks may reflect abnormal large-scale functional interactions between them. The FC changes between AN and mVN at baseline may be a compensatory effect caused by tinnitus, as phantom auditory sensations also activate visual areas (Zhou et al., 2019). However, after sound therapy, we did not find any network-level FC changes between the AN and VN; meanwhile, we found decreased level (i.e., positive) and increased levels (i.e., negative) internetwork FC between the DMN and AN in patients when compared with the HCs or patients at baseline. The DMN, including the medial prefrontal cortex and anterior cingulum (cingulate) cortex as well as the posterior cingulum (cingulate) cortex and precuneus, is associated with both cognitive and emotional control (Whitfield-Gabrieli et al., 2011). Moreover, it is most active at rest and shows reduced activity when a subject enters a task-based state involving attention or goal-directed behavior (Shulman et al., 1997). The DMN connectivity changes in our study after treatment were consistent with some studies that reported DMN dysfunction (Schmidt et al., 2013; Lanting et al., 2016). Lanting et al. (2016) believed that the DMN somehow plays a role in “hearing” internally generated sound (whether it is meaningful, e.g., in schizophrenia patients, or meaningless, e.g., in tinnitus patients).

Combined with previous studies, these findings indicated that significant differences exist in the abnormal changes in auditory-related brain structure and function before and after the narrow band sound therapy (compared with HCs or patients at baseline).

### Non-Auditory-Related Brain Structural and Functional Alterations Between the Patients and HCs and Between the Patients Before and After Sound Therapy

In addition to the auditory-related structural and functional changes, we also observed a significant increase in WMV in the cingulum (cingulate), right calcarine, left rolandic operculum, and the left parietal and frontal lobes in the patients after treatment compared with baseline. The Cingulum (cingulate) plays a large role in several large-scale networks in which tinnitus is involved; that is, tinnitus emerges as a function of several large-scale networks that bind together many aspects of salience, memory, perception, distress, and audition (De Ridder et al., 2011; Ridder et al., 2011; Schecklmann et al., 2012; Meyer et al., 2016). We believe that the increase in WMV in the cingulum (cingulate) indicates that tinnitus is closely related to dysfunction of the limbic system, which is in line with one of our prior studies (Chen et al., 2020). The calcarine cortex is an important part of the primary visual cortex, and it is the main relay station that transfers the signals coming from the retina. Some studies have shown that there is a close or even direct connection between the auditory and visual regions/subnetworks (Iurilli et al., 2012; Ibrahim et al., 2016). Thus, changes in the calcarine cortex may result from patients attending to phantom auditory sensations and having the visual areas contemporaneously activated (Zhou et al., 2019). Additionally, we found increased WMV in the rolandic operculum, which may correlate with tinnitus-related distress (Krick et al., 2015). Additionally, Job et al. (2012) found overactivity in the rolandic operculum; they speculated that this region was associated with middle ear proprioception, and changes in this region may suggest a hypothesis that tinnitus could arise as a proprioceptive illusion associated with widespread emotional and somatosensory dysfunction. Meanwhile, we found an increase in WMV in the left parietal and frontal lobes as the main parts of the frontoparietal network (FPN), and we believe that these areas play a large role in the process of decision-making and cognitive control (Vincent et al., 2008).

In addition to these changes in brain white matter, we also found significant differences from before to after sound therapy in the interactions among several RSNs that are not directly related to auditory function. For instance, at baseline, we found increased (i.e., less negative) and decreased (i.e., positive) internetwork FC between the mVN and the SMN and lVN, respectively. The VN and SMN are two independent systems that separately process visual and sensorimotor functions and play a role in the limbic system. A previous study on stroke suggested that the significantly changed internetwork FC between the two networks may reflect abnormal large-scale functional interactions among functional networks (Wang et al., 2014). Additionally, the vision network is divided into the mVN and lVN. The mVN includes primary visual areas, while the lVN encompasses non-primary regions of the visual cortex (Beckmann et al., 2005). We speculated that because of the increased compensatory stimulation between the auditory and visual networks caused by tinnitus, the functional interaction within the visual network is weakened. Although the exact mechanism underlying abnormal internetwork FC remains unclear, it may be the result of impairments in the thalamus, as mentioned above (Lv et al., 2020). After the treatment, we found decreased (i.e., positive) internetwork FC between the LFPN and DAN and increased (i.e., negative) internetwork FC between the LFPN and RFPN and among the LFPN, DMN, and DAN. According to a previous ICA study, the FPN is a lateralized network and has been commonly identified as an independent RSN (Smith et al., 2009). It primarily consists of two main parts: the dorsolateral prefrontal cortex and the posterior parietal cortex (several cognition/language areas), and it supports decision-making and cognitive control functions (Vincent et al., 2008). Few studies have reported tinnitus-related changes in the LFPN or RFPN (Lanting et al., 2016). Studies on stroke have proven that weakened connectivity of the FPN may represent a functional disconnection (Nomura et al., 2010) in brain regions that may underlie the cognitive impairments observed in these patients (Stebbins et al., 2008; Gottesman and Hillis, 2010). Therefore, the increased or decreased internetwork FC among the LFPN, RFPN, and DAN indicated that after tinnitus, the brain activity and functional connections in brain regions related to executive control, advanced cognition, and language are in an abnormal or dysfunctional state when compared with baseline. Meanwhile, we also observed increased internetwork FC between the DMN and DAN. The DAN is involved in visual attention (Gitelman, 2003), and the increased FC in our study was consistent with Schmidt et al. (2013), as they suggested that this increase in FC could be a compensatory attempt to handle the phantom stimulus, by delegating that process to non-attention-processing regions, such as the limbic system (Golm et al., 2013; Ooms et al., 2013).

These results suggest that in addition to auditory-related brain reorganization, there were also significant differences in the abnormal changes in non-auditory-related brain reorganization before and after sound therapy (narrow band noise). However, in the present study, we failed to find any intranetwork FC changes in tinnitus patients before and after sound therapy, which may have been due to the small sample size. Studies with a larger sample size are needed in the future. Although patients in this study with different sided tinnitus, the brain changes we found were only seen on the left side in some regions and on the right side in other areas, which is consistent with some previous studies (Husain et al., 2011; Chen et al., 2017, 2020; Liu et al., 2018; Besteher et al., 2019; Han et al., 2019a). We speculated the reason may be that many brain areas can be divided into several subregions based on their functions and the function of the right or left side of some regions in the brain is different while the exact mechanism is still not very clear.

### Limitations

There are several limitations in our study. First, the sample size of our study was relatively small, and we recruited only right-handed subjects. Second, as a longitudinal study, we scanned the HCs only once. In future studies, we need to follow up with the HCs for the same period (6 months) as the patients and scan them twice (at baseline and after 6 months of follow-up). Third, in this study, we recruited only tinnitus patients without HL and HCs. We will recruit tinnitus patients with and without HL in future studies. Fourth, we didn’t apply any sham therapy on patients and HCs, which we will apply to them in future studies. Fifth, the definition of tinnitus patients without HL was that there is no HL in the generally recognized frequencies (250–8 kHz: 0.250, 0.500, 1, 2, 3, 4, 6, and 8 kHz); thus, we cannot eliminate the possibility of hidden or slight HL in other frequency ranges currently.

## Conclusion

In conclusion, we found that significant differences exist in auditory-related and non-auditory-related brain functional and structural alterations in tinnitus patients without HL before and after sound therapy (narrowband stimulation), especially changes in white matter and internetwork FC, although the outcome of all the patients may not be very good after the treatment. Therefore, we supposed that sound therapy, especially the narrow band noise, may have a significant effect on brain reorganization in patients with idiopathic tinnitus without HL. It may advance the understanding of the neural pathophysiological mechanisms of idiopathic tinnitus after sound therapy.

## Data Availability Statement

The datasets generated for this study are available on request to the corresponding author.

## Ethics Statement

The studies involving human participants were reviewed and approved by the Institutional Review Board (IRB) of Beijing Friendship Hospital, Capital Medical University. The patients/participants provided their written informed consent to participate in this study. Written informed consent was obtained from the individual(s) for the publication of any potentially identifiable images or data included in this article.

## Author Contributions

QC was responsible for (1) study conception; (2) the acquisition, analysis, and interpretation of data; (3) drafting of the manuscript; (4) final approval of the version of the manuscript to be published; and (5) agreement to be accountable for all aspects of the work. ZDW, XW, PZ, ZY, and SG were responsible for (1) data analysis; (2) final approval of the version of the manuscript to be published; and (3) agreement to be accountable for all aspects of the work. HL was responsible for (1) revising the manuscript; (2) final approval of the version of the manuscript to be published; and (3) agreement to be accountable for all aspects of the work. ZCW was responsible for (1) study design; (2) manuscript revision; (3) final approval of the version of the manuscript to be published; and (4) agreement to be accountable for all aspects of the work. All authors contributed to the article and approved the submitted version.

## Conflict of Interest

The authors declare that the research was conducted in the absence of any commercial or financial relationships that could be construed as a potential conflict of interest.

## Footnotes

1 http://www.neuro.uni-jena.de

2 https://www.fil.ion.ucl.ac.uk/spm

3 http://www.rfmri.org/dpabi

4 http://mialab.mrn.org/software/gift/

5 https://www.nitrc.org/projects/mricron

6 http://www.nitrc.org/projects/bnv/

7 https://www.alivelearn.net/xjview/

## Abbreviations

HL: hearing loss
VBM: voxel-based morphometry
ICA: independent component analysis
HC: healthy control
FC: functional connectivity
CBT: cognitive behavioral therapy
CI: cochlear implant
THI: tinnitus handicap inventory
CSF: cerebrospinal fluid
GMV: gray matter volume
WMV: white matter volume
MNI: Montreal Neurological Institute
RSN: resting-state network
ROI: region of interest
MTG: middle temporal gyrus.
